# Compatibility and antimicrobial activity of silver nanoparticles synthesized using *Lycopersicon esculentum* peels

**DOI:** 10.1186/s13568-024-01774-5

**Published:** 2024-11-05

**Authors:** Esraa Ali, Samah H. Abu-Hussien, Esraa Hesham, Shimaa Ahmed, Habiba Mostafa, Ahmed Gamal, Salwa M. El-Sayed, Bahaa Hemdan, Ashraf Bakry, Naglaa M. Ebeed, Hesham Elhariry, Ahmed Galal, Basma T. Abd-Elhalim

**Affiliations:** 1 New Programs, Faculty of Agriculture, Ain Shams University, PO Box 68, Hadayek Shoubra, Cairo, 11241 Egypt; 2 Department of Agricultural Microbiology, Faculty of Agriculture, Ain Shams University, PO Box 68, Hadayek Shoubra, Cairo, 11241 Egypt; 3 Department of Biochemistry, Faculty of Agriculture, Ain Shams University, PO Box 68, Hadayek Shoubra, Cairo, 11241 Egypt; 4 Environmental and Climate Change Research Institute, National Research Center, Giza, 1266 Egypt; 5 Department of Genetics, Faculty of Agriculture, Ain Shams University, PO Box 68, Hadayek Shoubra, Cairo, 11241 Egypt; 6 Department of Food Science, Faculty of Agriculture, Ain Shams University, PO Box 68, Hadayek Shoubra, Cairo, 11241 Egypt; 7 Department of Poultry Production, Faculty of Agriculture, Ain Shams University, PO Box 68, Hadayek Shoubra, Cairo, 11241 Egypt

**Keywords:** Antimicrobial activity, Cytotoxicity, Half-maximal inhibitory concentration (IC_50_), Inhibition zone diameter, *Lycopersicon esculentum*, Silver nanoparticles

## Abstract

Nanoparticles have gained worldwide attention as a new alternative to chemical control agents due to their special physiochemical properties. The current study focused on the environmentally friendly synthesis of silver nanoparticles (AgNPs) using *Lycopersicon esculentum* peel. In addition to studying the intrinsic cytotoxic effectiveness of Le-AgNPs contribute to their antibacterial, and antifungal activities and the effect of nanoparticles on the integrity of their morphological behavior. The initiative biosynthesis of *L. esculentum* silver nanoparticles (Le-AgNPs) was indicated by the color change of *L. esculentum* (Le) extract mixed with silver nitrate (AgNO_3_) solution from faint pink to faint brown. UV–visible spectroscopy, Dynamic light scattering (DLS), Fourier-transform infrared spectroscopy, high-resolution transmission electron microscopy (HR-TEM), and X-ray diffraction techniques were used to characterize biosynthesized Le-AgNPs. Results of UV–visible spectroscopy recorded surface plasmon resonance at 310 nm for SPR of 2.5. The DLS results showed particles of 186 nm with a polydispersity index of 0.573. The FTIR spectrum indicated the existence of carboxyl, hydroxyl, phenolic, and amide functional groups. The HR-TEM analysis revealed quasi-spherical crystal particles of Le-AgNPs. Le-AgNPs had a negative zeta potential of − 68.44 mV, indicating high stability. *Bacillus subtilis* ATCC 6633 and *Escherichia coli* ATCC 8739 were the most susceptible pathogens to Le-AgNPs inhibition, with inhibition zone diameters (IZDs) of 4.0 and 0.92 cm, respectively. However, *Listeria monocytogenes* NC 013768 and *Shigella sonnei* DSM 5570 were the most resistant pathogens, with IZDs of 0.92 and 0.90 cm, respectively. Le-AgNPs demonstrated good inhibitory potential against pathogenic fungi, with IZDs of 3.0 and 0.92 cm against *Alternaria solani* ATCC 62102 and *Candida albicans* DSM 1386, respectively. The cytotoxicity effect was observed at a half-maximal inhibitory concentration (IC_50_) of 200.53 μg/ml on human colon NCM460D normal cells.

## Key points

- Le-AgNPs exhibit strong antimicrobial activity, which has a significant impact on a variety of pathogenic bacteria.
- Le-AgNPs have a bactericidal/fungicidal mode of action for various pathogenic bacteria and fungi.
- Le-AgNPs at concentrations below 200.53 μg/ml are safe and tolerated by normal human colon cells (NCM460D).

## Introduction

Nowadays, the need to discover novel biological antimicrobial compounds to eliminate many pathogenic microorganisms is increasing. Pathogenic microorganisms pose a global threat to public health due to their association with nosocomial infections (Frieri et al. 2017; Jan et al. 2021; Abdullah et al.., 2023). Pathogen spread into the environment is a critical development that is linked to increased morbidity, mortality, healthcare costs, and inappropriate antibiotic use (Mansoor et al. 2021; Murugaiyan et al. 2022). Resistance of microorganisms to various antimicrobial agents has become a serious issue (Mostafa et al. 2021). As a result, finding alternative biological sources is critical to avoiding the development of antimicrobial resistance (Murugaiyan et al. 2022).

The promising active compounds that could help resolve the mentioned problem are nanoparticles. Nanoparticles are particles with a diameter between 1 and 100 nm. They exhibit auspicious physiochemical properties, making them effective antimicrobial, anti-tumor, and anti-inflammatory agents (Mohanta et al. 2023). Nanoparticles are fabricated using a variety of techniques, including physical, chemical, and biological. Chemical and physical techniques produced many hazardous residues that harmed the safety of the nanoparticles; biological methods, on the other hand, are safer. Bottom-up chemical processes include solvothermal, co-precipitation, sonochemistry, pyrolysis, and green approaches; top-down processes include pulsed laser ablation, spray pyrolysis, and ball milling (Hamida et al. 2020). Conventional methods, like chemical treatments and top-down strategies, are labor-intensive, ineffective, and polluting. It has been demonstrated that these risks can be removed through biological methods, such as the use of microorganisms, plants, or their leftovers (Zia et al. 2017; Carbone et al. 2020).

Many plant residues and wastes are now being used as a source of nanoparticle reduction and biosynthesis agents because they are environmentally friendly, inexpensive, safe, and abundant (Chand et al. 2019). There are efforts underway to produce synthetic technologies mediated by plants that have a reduced environmental effect. Eco-friendly production procedures for nanoscale materials have been the focus of nanomaterials scientists in recent years. These particles' distinct physiochemical characteristics render them valuable across a wide range of disciplines, including medicine, optics, mechanics, photoelectrochemical sciences, and the chemical industry, where they have been employed in the production of nonlinear optical devices, drug-gene delivery systems, and catalysis (Si et al. 2020; and Kumar et al. 2021).

Green nanoparticle synthesis, particularly with plant extracts, is a relatively new ecologically friendly chemical technique that is straightforward, non-toxic, and reasonably priced (Carbone et al. 2020). Numerous phytoconstituents found in plants, including phenol, flavonoids, polysaccharides, alkaloids, terpenoids, carbohydrates, protein, lipids, fixed oil, and volatile oil, serve as a reducing and stabilizing agent for gold nanoparticles (Faisal et al. 2022). The active component in the plant has a major role in regulating the size and synthesis of the generated nanoparticles. For this reason, choosing the right plants to produce nanoparticles is crucial. One of the most famous and abundant plant residues is *L. esculentum,* which is well known for its high levels of nutrients with anticancer, antimutagenic, antiangiogenic, antioxidant, antibacterial, and antiviral properties (Al-Radadi et al. 2021). The utilization of plant extracts as capping and reducing agents has allowed for the environmentally benign synthesis of a variety of nanoparticles, including Fe, Ag, Ni, Sb, Ca, Au, Cu and Zn (Hussain et al. 2022). The use of silver in traditional medicine confirms the long-known antimicrobial activity of silver nanoparticles (Al-Radadi et al. 2022).

The antibacterial, antifungal, and antiviral characteristics of silver nanoparticles are extensive. By penetrating bacterial cell walls, AgNPs can alter the structure of cell membranes and perhaps cause cell death. Their enormous surface area-to-volume ratio and nanoscale size both contribute to their effectiveness. They can release silver ions, create reactive oxygen species, and make cell membranes more permeable. They can also stop the replication of deoxyribonucleic acid (Shah et al. 2022).

AgNPs were reported to have antimicrobilal effects against *Enterococcus faecium, Staphylococcus aureus, Klebsiella pneumoniae, Acinetobacter baumannii, Pseudomonas aeruginosa*, and *Enterobacter cloacae*. According to (Chand et al. 2019; Si et al. 2020). *L. esculentum* plays an important role in the biosynthesis of silver and gold nanoparticles through the use of callus and leaf extracts with good antimicrobial activity against *Bacillus subtilis, Candida albicans, Proteus vulgaris, Pseudomonas sp., and Staphylococcus aureus* (Kumar et al. 2021). Antifungal properties of TeaNPs were examined on *C. auris* and *C. neoformans*.

So the current study aimed to biosynthesize and characterize AgNPs using *L. esculentum* peel extract. The characterization uses UV–visible spectroscopy, Dynamic light scattering (DLS), Fourier-transform infrared (FTIR) spectroscopy, high-resolution transmission electron microscopy (HR-TEM), and X-ray diffraction (XRD) techniques. As well as investigate their cytotoxic effect and inhibitory potential against pathogenic bacteria and fungi to be applied in the future in many fields such as agricultural, pharmaceutical, and food industries.

## Materials and methods

### Chemicals and reagents

Silver nitrate (AgNO_3_) was purchased from Sigma Aldrich, Germany. Nutrient, malt, and Muller-Henton media were purchased from Oxoid, India. Ampicillin, Streptomycin, and Fluconazole (1000 µg/ml) were obtained from Elnasr Pharma, Cairo, Egypt. MTT dye (3-(4,5-dimethylthiazol-2-yl)-2,5-diphenyltetrazolium bromide) was obtained from Bio Basic Inc., Canada. All chemicals are analytical grades.

### *L. esculentum* peels collection

*L. esculentum* peel was collected in plastic bags from a local restaurant in Cairo, Egypt, and quickly transported using an ice box to the microbiology lab at the Faculty of Agriculture, Ain Shams University, Cairo, Egypt. Peels were kept at 4 °C until further use.

### *L. esculentum* peels aqueous extract (LPAE) preparation

The LPAE was prepared in the following manner: All *L. esculentum* peels were washed three times with deionized water (DW). Twenty grams of *L. esculentum* peels were blended in 80 ml of deionized water using an electric blender (Moulinex, France). After that, the blended *L. esculentum* peels were kept in the dark for 24 h. The obtained mixture was then filtered through filter paper (Whatman No. 1) (Carbone et al. 2020). The entire filtrate (aqueous extract) was collected in screw tubes and stored at 4 °C for future studies.

### Source of pathogenic strains

Eleven pathogenic strains (6 bacteria and 5 fungi) were collected from the Agric. Microbiology Department, Faculty of Agriculture, Ain Shams University in Cairo, Egypt. The pathogens collected were *B. subtilis* ATCC 6633, *E. coli* ATCC 8739, *K. quasipneumoniae* ATCC 700603, *S. typhi* DSM 17058, *S. sonnei* DSM 5570, *L. monocytogenes* NC 013768, *A. solani* ATCC 62102, *A. flavus* ATCC 9643, *C. albicans* DSM 1386, *F. oxysporum* ATCC 62506, and *R. oryazae* ATCC 96382.

### Standard inoculum

Standard inoculum for all tested bacteria was prepared by picking up a pure single colony of each tested bacterial culture and inoculating it into a conical flask (250 ml) containing 50 ml of nutrient broth medium** (**Galal et al. 2021**)**. After inoculation, all cultures were incubated for 24 h at 37 °C and 150rpm on a rotary shaker (Shin Saeng, South Korea). One milliliter of the cultures contained 4.5–5.77×10^5^ CFU/ml. Standard inoculum for fungi was prepared by scratching their growth on malt agar slants in 10 ml of sterilized water and collecting their spore suspensions. The collected spore suspensions contained 1.1–1.3×10^8^Spore/ml.

### Biosyntheis of Le-AgNPs

Le-AgNPs biosyntheis was performed by adding 20 ml of LPAE to 80 ml of the 1 mM AgNO_3_ solution. The mixture was vigorously mixed and stored overnight on a rotary shaker (Shin Saeng, South Korea) at 150rpm and 30 °C in the dark to minimize photoactivation of AgNO_3_. The reduction of Ag+ ion to Ag0 was initially confirmed by the color change from pale pink to pale brown is a positive result. Followed by filtration through filter paper (Whatman No. 1) to get rid of any impurities and the obtained filtrate was used in the assessment of nanoparticles formation and characterization. For further investigations the obtained solution was centrifuged (SIGMA 2-16 P centrifuge) at 10,000rpm for 5 min. The supernatant was discarded and the pellet obtained was washed three times with distilled water. The purified air dried AgNPs were used for the subsequent characterization studies (Murugaiyan et al. 2022).

### Characterization of Le-AgNPs

The biofabrication of Le-AgNPs was demonstrated by measuring SPR absorbance with UV–Vis spectroscopy (JASCO Corp., V-570) at wavelength ragnged betweem 200–700 nm **(**Murugaiyan et al. 2022**)** at Egyptian petroleum research institute (EPRI), Cairo, Egypt. The morphology, shape, and agglomeration images of Le-AgNPs were obtained using the HR-TEM) JEOL, and JEM-2100, Japan) at Medical and Scientific Centre of Excellent, National Research Center (NRC), Cairo, Egypt, and operated at 200 kV with a counting rate of 2838 cps. The image resolution is 512 by 442, with a pixel size of 0.04 μm. The accelerating voltage is 20.0 kV (Mohanta et al. 2023). Le-AgNPs were analyzed using Fourier transform infrared spectroscopy (FTIR) (Bruker VERTEX 80, Germany) at “CEB” “Creative Egyptian Biotechnologists” Company, Giza, Egypt with a wave number range of 4000–400 cm^−1^, resolution of 4 cm^−1^, and refractive index of 2.4 (Mohamed et al. 2017; Carbone et al. 2020). The concentration of biosynthesized Le-AgNPs in the solution mixture was determined using atomic absorption spectroscopy (AAS) (Thermo Scientific, ICE3000 SERIE, USA) at "CEB''Creative Egyptian Biotechnologists'' Company, Giza, Egypt. The dynamic light scattering (DLS) was estimated to determine size of Le-AgNPs using the distribution Zetasizer Nano Series (HT), Nano ZS, Malvern Instruments, UK (Zia et al. 2017) at Egyptian petroleum research institute (EPRI), Cairo, Egypt. Zeta potential, a characteristic of charged colloids, was measured using phase analysis light scattering based on DLS and laser Doppler velocimetry, with a count rate of 286 kHz, wavelength (λ = 1.54 nm) at 30 °C, and viscosity of 0.995 cp (Yap et al. 2020) at "CEB''Creative Egyptian Biotechnologists'' Company, Giza, Egypt. According to the Joint Committee on Powder Diffraction Standards JCPDS Card No. 36–1451 (Faisal et al. 2024) obtained at "CEB''Creative Egyptian Biotechnologists'' Company, Giza, Egypt.

### Antimicrobial susceptibility of Le-AgNPs using agar well-diffusion assay

To investigate the inhibitory potentials of Le-AgNPs, standard CLSI guidelines were followed using agar well diffusion method. Briefly, Muller-Hinton and malt agar media were poured into Petri dishes and left until solidified. One ml of all bacteria and fungi standard inocula were spread individually onto the solidified Muller Hinton (MH) and malt plates for bacteria and fungi, respectively. Wells were made on the inoculated agar plates with a sterilized cork porer with a diameter of 0.9 cm. The wells were filled with 10 µl of each of the following: LPAE (control), Le-AgNPs with various concentrations between 1000–12.5 µg/ml, and standard antibiotics (1000 µg/ml) of Ampicillin for G^+ve^ bacteria, Streptomycin for G^−ve^ bacteria, and Fluconazole for fungi and yeasts. All inoculated plates were incubated at 37 °C and 28 °C for 24 and 72 h with bacteria and fungi, respectively. Inhibition zone diameter (IZD) was measured and recorded in centimeters (cm). The antimicrobial activity index (AAI) was calculated using the following equation **(**Galal et al. 2021;Abd-Elhalim et al. 2023**)**:

1 $$\text{Antimicrobial activity index }(\text{AAI}) =\frac{IZD \,of\, Le-AgNPs} {IZD\, of\, standard\, antibiotic}$$

### Minimum inhibitory concentration (MIC) of Le-AgNPs

The minimum inhibitory concentration (MIC) of Le-AgNPs was determined by reinoculation from free growth zones into MHA and malt agar media, then incubated at 37 °C and 28 °C for 24 h and 72 h with bacteria and fungi, respectively. After incubation, microbial growth was observed. The MIC was defined as the lowest concentration of Le-AgNPs that inhibited microbial growth (Galal et al. 2021; Ijaz et al. 2022; Abd-Elhalim et al. 2023).

### Assessment of minimum lethal concentration (MLC) of Le-AgNPs

MLC was determined by reinoculating the free growth zones of MIC on MHA and malt agar media and incubating at 37 °C and 28 °C for 24 and 72 h with bacteria and fungi, respectively. MLC was defined as the lowest concentration of Ag that inhibited microbial growth (Galal et al. 2021; Ijaz et al. 2022).

### Inhibitory action of Le-AgNPs

Le-AgNP inhibitory action was assessed according to the following equation:

2 $$Le-AgNPs\, inhibitory \,action=\frac{MLC \,value}{ MIC \,value}$$

Le-AgNPs is classified as bactericidal or fungicidal agents if the MBC or MFC/MIC value is ≥ 4. Bacteriostatic or fungistatic means the value is less than 2 (Galal et al. 2021; Ijaz et al. 2022; Abd-Elhalim et al. 2023).

### Scanning electron microscope (SEM) investigation for Le-AgNPs antimicrobial effect

An SEM was applied to study the antimicrobial effect of Le-AgNPs at Creative Egyptian Biotechnologists (CEB) in Giza, Egypt. A100 μl of bacterial growth preparation (1 × 10^6^ CFU/ml) were separated into two groups the first group was added to tubes containing 50 mM phosphate buffer solution (PBS) of pH 7.0 and the second group exposured to 1000 μg/ml Le-AgNPs then both sets incubated at 37 °C for 12 h at 150rpm. The bacterial cells were then harvested by centrifugation three times at 10,000rpm for 5 min at 4 °C and washed with 50 mM PBS (pH 7.0). The samples were then fixed with 3% glutaraldehyde in 50 mM PBS (pH 7.0) at room temperature for 4 h without agitation before being rinsed four times with PBS for 20 min. After fixation using 1% osmic acid at room temperature for 2 h**,** samples were washed with distilled water for 15 min. After dehydrating samples with a graded alcohol series of 30%, 50%, 70%, 80%, 90%, and 95%, repeat three times with 100% for 15 min in each series. Finally, samples were incubated overnight in isoamyl acetate. Samples were then dried with dry CO_2_ and gold-coated. Observation using a scanning electron microscope (S-3400N, SEM system, Hitachi, Tokyo, Japan) (Venkatesh et al. 2018).

### Biocombitability of Le-AgNPs against human *colon* NCM460D cells

The biocombitability of Le-AgNPs was assessed at Science Way Lab for Scientific Services, Cairo, Egypt using an MTT assay. Normal human colon NCM460D cell tissue culture (1 × 10^5^ cells/ml (100 µl/well)) was inoculated in 96-well microtiter plates containing Roswell Park Memorial Institute (RPMI) growth medium and then incubated at 37 °C for 24 h to obtain a complete monolayer sheet. The growth medium was discarded, and the cells were washed twice with phosphate buffer solution (PBS) PH 7.0. The tested sample was diluted (two-fold dilutions) in RPMI medium with 2% serum maintenance medium to obtain concentrations of 1000 (control), 500, 250, 125, and 62.25 µg/ml. From each dilution, 0.1 ml was tested in different wells, with three wells serving as a control, and incubated at 37 °C. After incubation, prepare a 5 mg MTT dye solution in 1 ml of PBS and add 20 µl of the prepared solution to each well. The microplate was then placed on a shaking table at 150rpm for 5 min to mix the MTT with the cells before being incubated at 37 °C in 5% CO_2_ for 4 h to allow the cells to metabolize the MTT dye. Finally, the excess MTT solution was removed, and the plates were allowed to dry. The MTT dye's metabolic product (formazan) was resuspended in 200 µl of dimethylsulfoxide solvent (DMSO). The plate was then shaken at 150 rpm for 5 min to thoroughly mix the formazan into the DMSO. The optical density was measured at 560 nm and subtracted from the background at 620 nm. Optical density (O.D.) is directly proportional to cell quantity. Cell morphology changes were observed and noted using a phase microscope (MML1200, Germany) (van de Loosdrecht et al. 1994).

### Statistical analysis

All collected data were statistically analyzed using IBM® SPSS® Statistics software (2017). Duncan's test at a *P*-value of 0.05 was applied (Duncan 1955). The IC_50_ values were calculated using GraphPad Prism 8.4.1 (GraphPad Software, San Diego, CA, www.graphpad.com) as mean ± SD (n = 3). UV–VIS and FTIR absorption spectra of LPAE and Le-AgNPs were plotted using OriginPro 2022 (64-bit) SR1 v9.9.0.2 software https:// www. Origi nlab. com/ index. aspx? go = Suppo rt& pid = 4440. XRD spectra of Le-AgNPs was plotted using Profex 2019 (64-bit) v5.1.1.

## Results

### Biosynthesis of Le-AgNPs

**Fig. 1:**
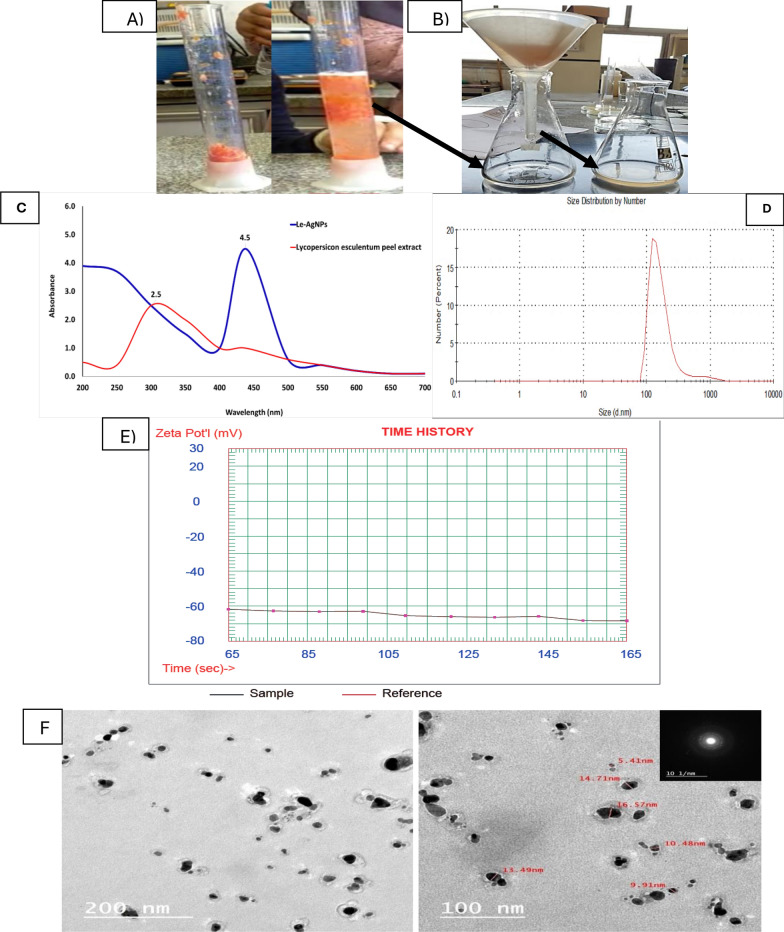
**A** Preparation of *L. esculentum* peel extract (LPAE) (control); **B***L. esculentum* peels extract-AgNO_3_ mixture before and after biosynthesis of Le-AgNPs. **C** Uv–vis spectroscopy characterization of Le-AgNPs and LPAE (control). **D** Dynamic light scattering (DLS) investigation of Le-AgNPs fabricated using LPAE. **E** Zeta potential characterization of Le-AgNPs fabricated using LPAE. **F** High resolution transmission electron microscope (HR-TEM) investigation of Le-AgNPs fabricated using *L. esculentum* peels extract

After preparation of *L. esculentum* peels extract (LPAE) (Fig. 1A), the Le-AgNPs biosynthesis process was primarily indicated by a change in the mixture of LPAE and AgNO_3_ color from pale pink to pale brownish due to the reduction of Ag^+^ to Ag^0^, as illustrated in Fig. 1B.

### Characterization of the biosynthesized Le-AgNPs

#### UV–Vis spectroscopy analysis

Figure 1C shows UV–visible spectrum data for a strong peak at 438 nm with an SPR of 4.5 which corresponded to Le-AgNPs. In contrast, LPAE at 310 nm for an absorbance units (a.u.) of 2.5 which is unrelated to AgNPs formation.

#### DLS analysis

The DLS examination showed that the Le-AgNPs particles have a size of 186 nm with polydispersity index (PDI) of 0.578 (Fig. 1D).

#### Zeta potential

As mentioned in Fig. 1E, Le-AgNPs have a negative zeta potential of -68.44 mV. The phytochemicals in the LPAE appear to be responsible for the AgNPs' capping action, which is why ξ has a negative value.

### High-resolution transmission electron microscopy (HR-TEM)

As shown in Fig. 1F Le-AgNPs were quasi-spherical shape, with particle sizes ranging from 5.14–16.57 nm. It also can be noticed from the TEM images that the biosynthesized AgNPs are well stabilized and capped by active compounds present in LPAE.

### Fourier transform infrared spectroscopy (FTIR) analysis

**Fig. 2:**
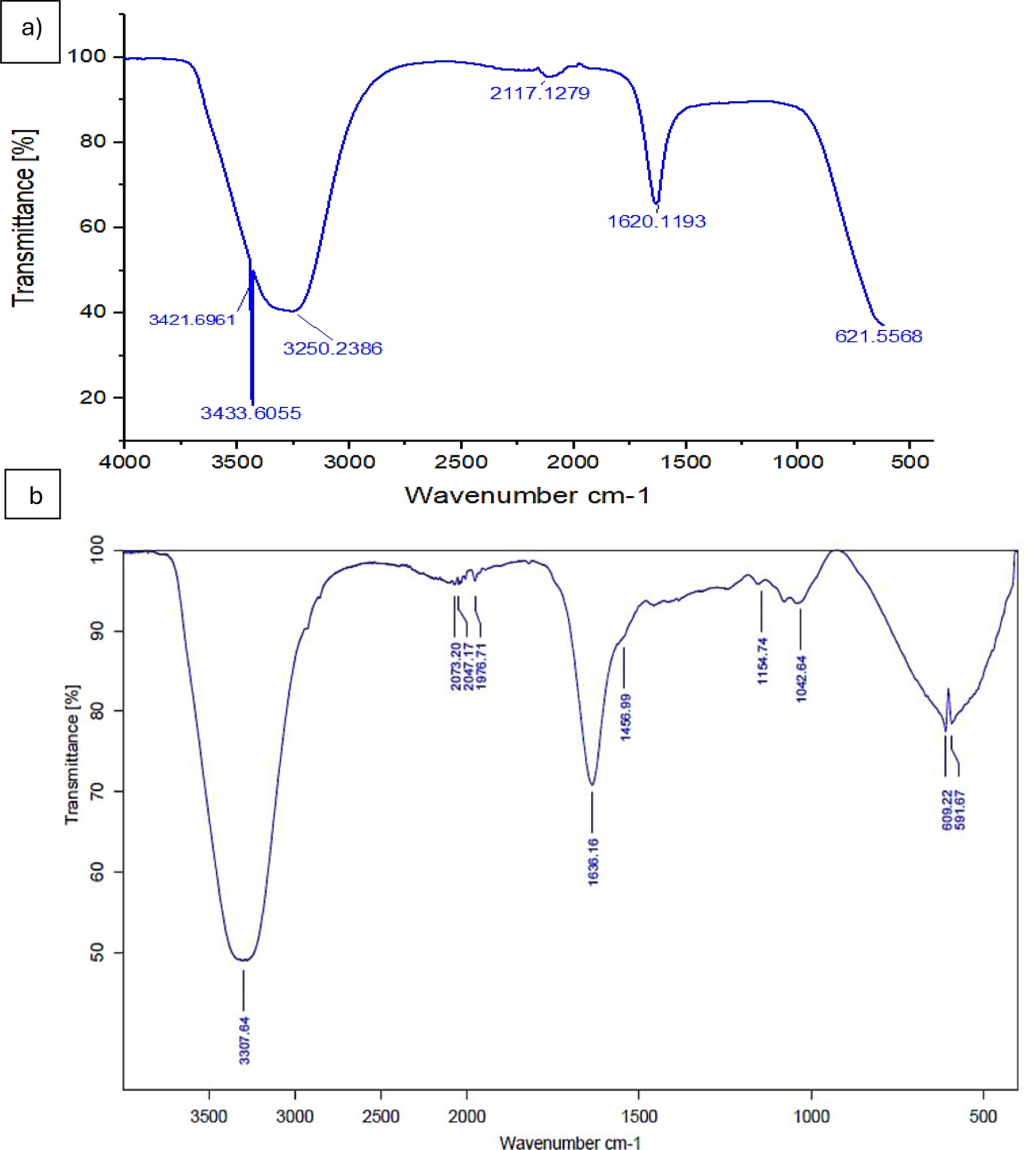
FTIR absorption spectra of **A** *L. esculentum* peels extract (control), and **B** FTIR absorption spectra of Le-AgNPs fabricated using *L. esculentum* peels extract

FTIR analysis was performed to characterize active functional groups that correspond to the biofabrication and stabilizing process of Le-AgNPs. Figure 2A shows six spectrum peaks in LPAE (control) at 3433.60, 3421.96, 3250.23, 2117.12, 1620.12, and 621.55 cm^−1^ corresponding to O–H stretching of alcohol, O–H stretchingof carboxylic acid, N=C=S stretching of isothiocyanate, N–H bending of amine, and C–Cl stretching of halo compound, respectively. In the infrared spectrum of the Le-AgNPs solution (Fig. 2B), ten independent peaks were detected. This spectrum reveals a strong bands at 3307.64, 2073.20, 2047.17, 1976.61, 1636.16, 1456.99, 1154.74, 1042.64, 609.22 and 591.67 cm^−1^. The bands corresponding to N–H stretching of aliphatic primary amine, N=C=S stretching of isothiocyanate, C=C=N stretching of ketenimine, C-H bending of aromatic compound, C=O stretching of secondary amide, C–H bendingof alkane, C–N stretching of amine, CO–O–CO stretching of anhydride, C–Cl stretchingof halo compound, and C–I stretchingof halo compound, respectively. The formation of silver nanoparticles can be confirmed by the presence of a peaks at 609 and 1042.09 cm^−1^ belong to bending vibration of Ag.

### X-ray Diffraction (XRD) analysis

**Fig. 3:**
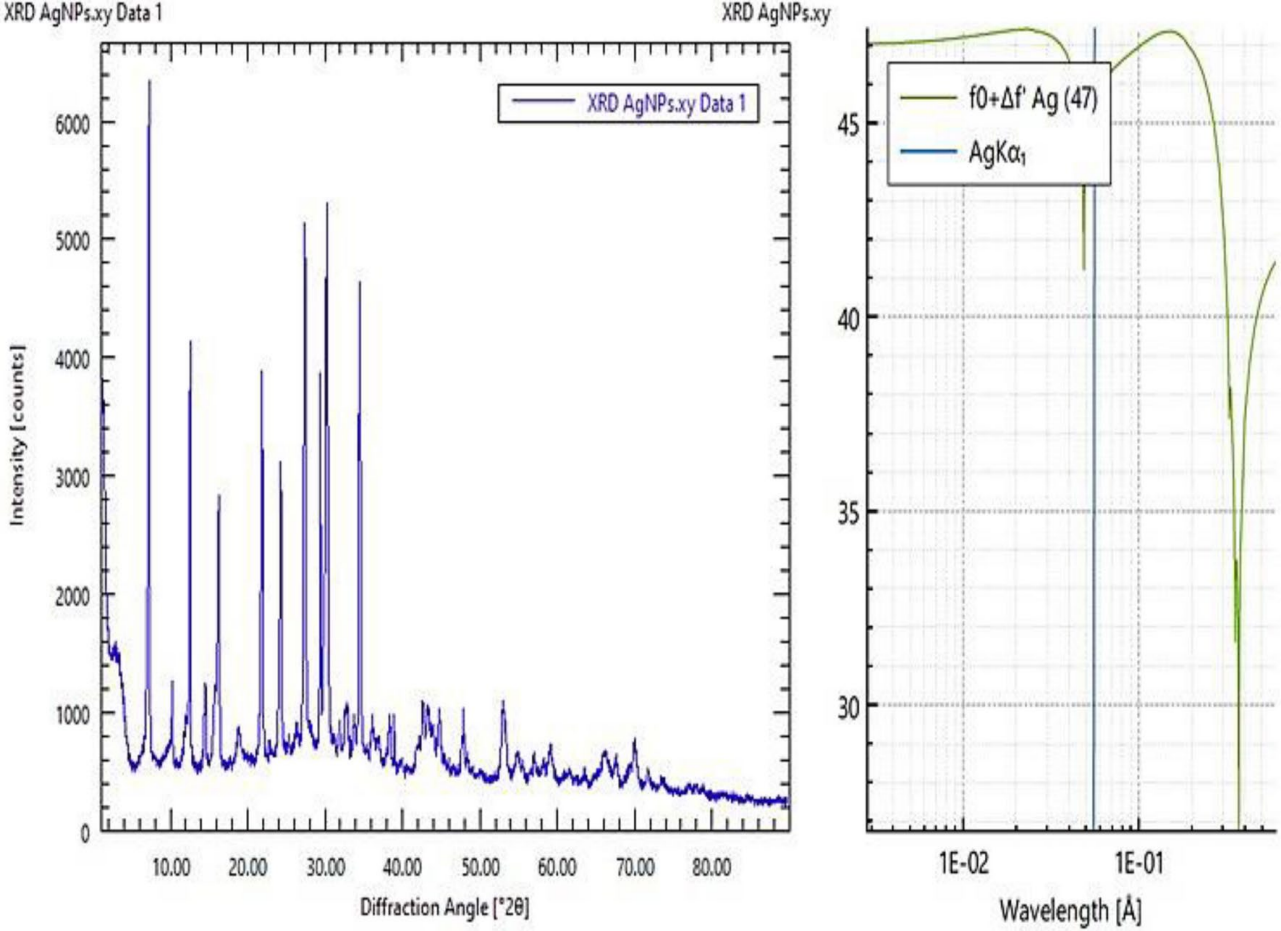
X-ray diffraction (XRD) analysis of Le-AgNPs fabricated using *L. esculentum* peels extract at diffraction angle range of 10.0–70.0° 2θ

The major observed XRD peaks are 6400, 4100, 1000, 2800, 1000, 3900, 3100, 5200, 4800, 5400, 1000, 1100, 950, 1100, and 900 at 2 Theta or diffraction angle of 7.96, 12.32, 14.24, 16.44, 18.32, 22.56, 24,54, 27.69, 30.12, 32.32, 38.24, 44.44, 48.32, 54.45, and 70.72° respectively as shown in Fig. 3. The distinct XRD reflection planes confirms the FCC (face-centered cubic structure) crystal morphology as confirmed by JCPDS Card No. 36–1451.

### Inhibitory activity of the biosynthesized Le-AgNPs

Results in Table 1 showed that all tested pathogenic bacteria were highly susceptible to Le-AgNPs, whereas the LPAE didn’t exhibit antimicrobial effect. It was also noticed that all fungi were resistant for all treatments. *B. subtilis*, *S. typhi*, and *S. sonii* were the most sensitive bacterial strains to the biosynthesized Le-AgNPs with inhibition zone diameters (IZDs) of 3.8, 2.6, and 2.6 cm, respectively. However, no antifungal activity index was observed for *A. solani* ATCC 62102*, A. flavus* ATCC 9643*, F. oxysporum* ATCC 62506, and * R. oryazae* ATCC 96382.

**Table 1:** Inhibitory activity and activity index (AI) of Le-AgNPs and standard antibiotics against pathogenic bacterial and fungal strains after incubation at 37 °C and 28 °C for 24 and 72 h, respectively

Pathogen strains	Inhibition zone diameter (cm)			Activity index (AI)
	SAb (1000 µg/ml)	LPAE (10 µl)	Le-AgNPs (1000 µg/ml)	
G + ve Bacteria				
*B. subtilis* ATCC 6633	4.0^a^ ± 0.86	0.00	3.8^b^ ± 0.41	0.95
*L. monocytogenes* NC 013768	0.92^g^ ± 0.24	0.00	1.1^f^ ± 0.55	1.19
G-ve Bacteria				
*E. coli* ATCC 8739	0.92^g^ ± 0.36	0.00	1.0^f^ ± 0.14	1.08
*K. quasipneumoniae* ATCC 700603	1.00^f^ ± 0.85	0.00	2.0^e^ ± 0.77	2.00
*S. typhi* DSM 17058	0.91^g^ ± 0.22	0.00	2.6^d^ ± 0.11	2.86
*S. sonnei* DSM 5570	0.90^g^ ± 0.24	0.00	2.6^d^ ± 0.30	2.88
Fungi and yeasts				
*A. solani* ATCC 62102	3.00^c^ ± 0.50	0.00	0.00	0.00
*A. flavus* ATCC 9643	2.80^d^ ± 0.82	0.00	0.00	0.00
*F. oxysporum* ATCC 62506	2.10^e^ ± 0.66	0.00	0.00	0.00
*R. oryazae* ATCC 96382	1.90^e^ ± 0.50	0.00	0.00	0.00
*C. albicans* DSM 1386	0.92^g^ ± 0.40	0.00	1.1^f^ ± 0.50	1.19

SAb = Standard antibiotics were Streptomycin, Ampicillin, and Fluconazole against G^+ve^ bacteria, G^−ve^ bacteria, and fungi, respectively. *Cm = Centimeter, AI = activity index, and SE (±)  = standard error. Values with the same letter do not significantly differ from each other, according to Duncan (1955), at a 5% level

### Minimum inhibition concentration (MIC) for Le-AgNPs

Results in Table 2 show the MIC values of Le-AgNPs (1000–12.5 μg/ml) against the tested pathogenic bacteria and fungi strains. The MIC value was 250 μg/ml for* E. coli*,* K. quasipneumoniae*,* S. typhi*, *L. monocytogenes*, and *S. sonnei,* while it was 75.0 μg/ml for *B. subtilis*. The Le-AgNPs demonstrated 100% antibacterial spectrum activity at concentrations ranging from 1000 to 250 μg/ml, but only 16.7% at 125 and 75 μg/ml. At concentrations ranging from 50 to 12.5 μg/ml, no antibacterial activity was observed. Table 2 shows that Le-AgNPs had antifungal activity against* C. albicans* with a MIC of 500 μg/ml, while other fungal strains had no antifungal effect. At concentrations ranging from 1000 to 500 μg/ml, all fungi and yeast strains showed 80.0% activity. Furthermore, concentrations of 250–12.5 μg/ml showed no activity against the tested fungi and yeast strains.

**Table 2:** Minimum inhibitory concentration (MIC) of Le-AgNPs against pathogenic bacterial and fungal strains after incubation at 37 °C and 28 °C for 24 and 72 h, respectively

Pathogenic bacteria	MIC (µg/ml) of Le − AgNPs							
	1000	500	250	125	75	50	25	12.5
*B. subtilis* ATCC 6633	−	−	−	−	−	+	+	+
*E. coli* ATCC 8739	−	−	−	+	+	+	+	+
*K. quasipneumoniae* ATCC 700603	−	−	−	+	+	+	+	+
*L. monocytogenes* NC 013768	−	−	−	+	+	+	+	+
*S. typhi* DSM 17058	−	−	−	+	+	+	+	+
*S. sonnei* DSM 5570	−	−	−	+	+	+	+	+
Spectrum of activity (%)	6/6	6/6	6/6	1/6	1/6	0/6	0/6	0/6
	100	100	100	16.7	16.7	0	0	0

Pathogenic fungi	MIC (µg/ml) of Le − AgNPs							
	1000	500	250	125	75	50	25	12.5
*A. solani* ATCC 62102	+	+	+	+	+	+	+	+
*A. flavus* ATCC 9643	+	+	+	+	+	+	+	+
*C. albicans* DSM 1386	−	−	+	+	+	+	+	+
*F. oxysporum* ATCC 62506	+	+	+	+	+	+	+	+
*R. oryazae* ATCC 96382	+	+	+	+	+	+	+	+
Spectrum of activity (%)	4/5	4/5	0/5	0/5	0/5	0/5	0/5	0/5
	80.0	80.0	0.00	0.00	0.00	0.00	0.00	0.00

−  = No growth, and +  = Positive growth

### Minimum lethal concentration (MLC) for Le-AgNPs

Values of minimum lethal concentration MLC (MBC and/or MFC) for Le-AgNPs are presented in Table 3. The MBC value was exhibited at 500 μg/ml for* E. coli*, *K. quasipneumoniae*,* S. typhi*, *L. monocytogenes*, and *S. sonnei*, while it was 125.0 μg/ml with *B. subtilis*. The results clearly showed 100% of the antibacterial spectrum activity of Le-AgNPs at concentrations ranging from 1000 to 500 μg/ml, whereas at concentrations of 250 and 125 μg/ml, the activity was 16.7%. However, concentrations of 75–12.5 μg/mL had no observed inhibitory activity. The MIC of Le-AgNPs against* C. albicans* was 1000 μg/ml, while the other fungal strains exhibited no antifungal effect, as shown in Table 3. At a concentration of 1000 μg/ml, 80.0% of the spectrum of activity was attained for all fungi and yeast strains. In addition, concentrations ranging from 500 to 12.5 μg/mL did not display any activity against the tested fungi and yeast strains.

**Table 3:** Minimum bactericidal and fungicidal concentration (MBC & MFC) of Le-AgNPs against pathogenic bacterial and fungal strains after incubation at 37° and 28 °C for 24 and 72 h, respectively

Pathogenic bacteria	MBC (µg/ml) of Le − AgNPs							
	1000	500	250	125	75	50	25	12.5
*B. subtilis* ATCC 6633	−	−	−	−	+	+	+	+
*E. coli* ATCC 8739	−	−	+	+	+	+	+	+
*K. quasipneumoniae* ATCC 700603	−	−	+	+	+	+	+	+
*L. monocytogenes* NC 013768	−	−	+	+	+	+	+	+
*S. typhi* DSM 17058	−	−	+	+	+	+	+	+
*S. sonnei* DSM 5570	−	−	+	+	+	+	+	+
Spectrum of activity (%)	6/6	6/6	1/6	1/6	0/6	0/6	0/6	0/6
	100	100	16.7	16.7	0.00	0.00	0.00	0.00

Pathogenic fungi	MFC (µg/ml) of Le − AgNPs							
	1000	500	250	125	75	50	25	12.5
*A. solani* ATCC 62102	+	+	+	+	+	+	+	+
*A. flavus* ATCC 9643	+	+	+	+	+	+	+	+
*C. albicans* DSM 1386	−	+	+	+	+	+	+	+
*F. oxysporum* ATCC 62506	+	+	+	+	+	+	+	+
*R. oryazae* ATCC 96382	+	+	+	+	+	+	+	+
Spectrum of activity (%)	4/5	0/5	0/5	0/5	0/5	0/5	0/5	0/5
	80.00	0.00	0.00	0.00	0.00	0.00	0.00	0.00

−  = No growth; +  = growth

### Le-AgNPs inhibitory action

Finally, it could be observed that the inhibitory action of Le-AgNPs against pathogenic bacterial and fungal strains is shown in Table 4. Results indicated that the Le-AgNPs have a bactericidal and fungicidal effect with MBC or MFC/MIC ≤ 2 toward 7 strains:*B. subtilis*, *E. coli*, *K. quasipneumoniae*, *L. monocytogenes*, *S. typhi*, *S. sonnei*, and* C. albicans.*

**Table 4:** Inhibitory action for Le-AgNPs using pathogenic bacterial and fungal strains after incubation at 37° and 28 °C for 24 and 72 h, respectively

Pathogenic bacteria	MIC (Le-AgNPs µg/ml)	MBC (Le-AgNPs µg/ml)	MBC/MIC ratio	Inhibition action
Bacteria				
*B. subtilis* ATCC 6633	75	125	1.7	+
*E. coli* ATCC 8739	250	500	2	+
*K. quasipneumoniae* ATCC 700603	250	500	2	+
*L. monocytogenes* NC 013768	250	500	2	+
*S. typhi* DSM 17058	250	500	2	+
*S. sonnei* DSM 5570	250	500	2	+
Fungi				
*A. solani* ATCC 62102	0	0	0	0
*A. flavus* ATCC 9643	0	0	0	0
*C. albicans* DSM 1386	500	1000	2	+
*F. oxysporum* ATCC 62506	0	0	0	0
*R. oryazae* ATCC 96382	0	0	0	0

Bactericidal/Fungicidal (+) =  ≤ 2 and Bacteriostatic/Fungistatic (−) effect =  ≥ 4

### SEM investigation of Le-AgNPs antimicrobial effect

**Fig. 4:**
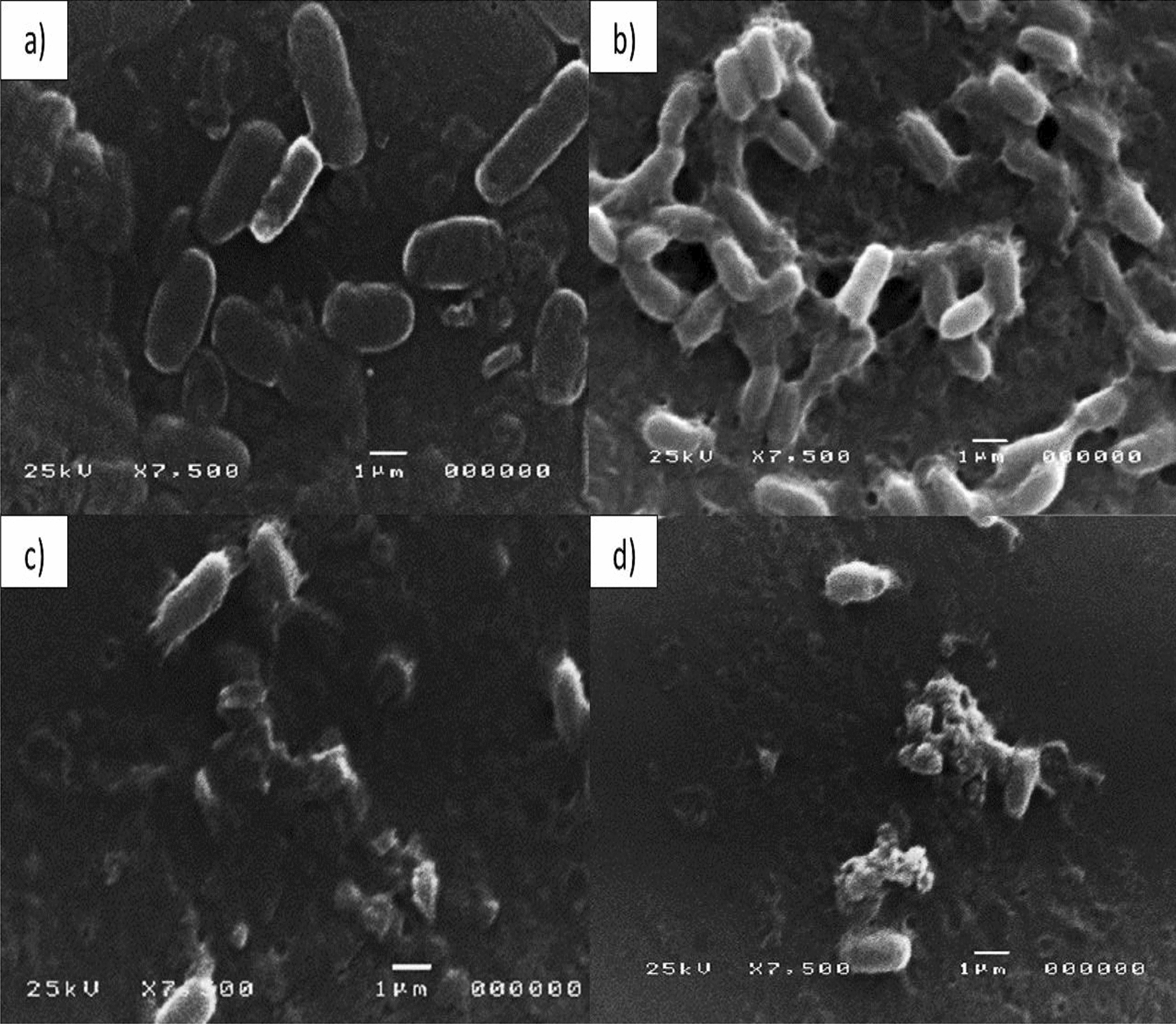
SEM images of antimicrobial investigation using **A** *Bacillus subtilis*, and **B***E. coli*, cells control treated with phosphate buffer (pH = 7.0), **C***Bacillus subtilis* treated with 1000 μg/ml Le-AgNPs, and **D***E. coli* cells treated with 1000 μg/ml Le-AgNPs

The Le-AgNPs antimicrobial action was confirmed with SEM investigation, as many clear different signs in cell morphology among control (Fig. 4A & B) and treated cells with damage and modifications to the whole cell or/and cell wall for the most affected microorganisms *B. subtilis* and *E. coli* with 1000 µg/mL Le-AgNPs. The cell changes included irregular cell surfaces, unusual morphological shapes, disrupted cell walls, and/or complete cell lacerations, as shown in Fig. 4C & D.

### Biocombitability of Le-AgNPs

**Fig. 5:**
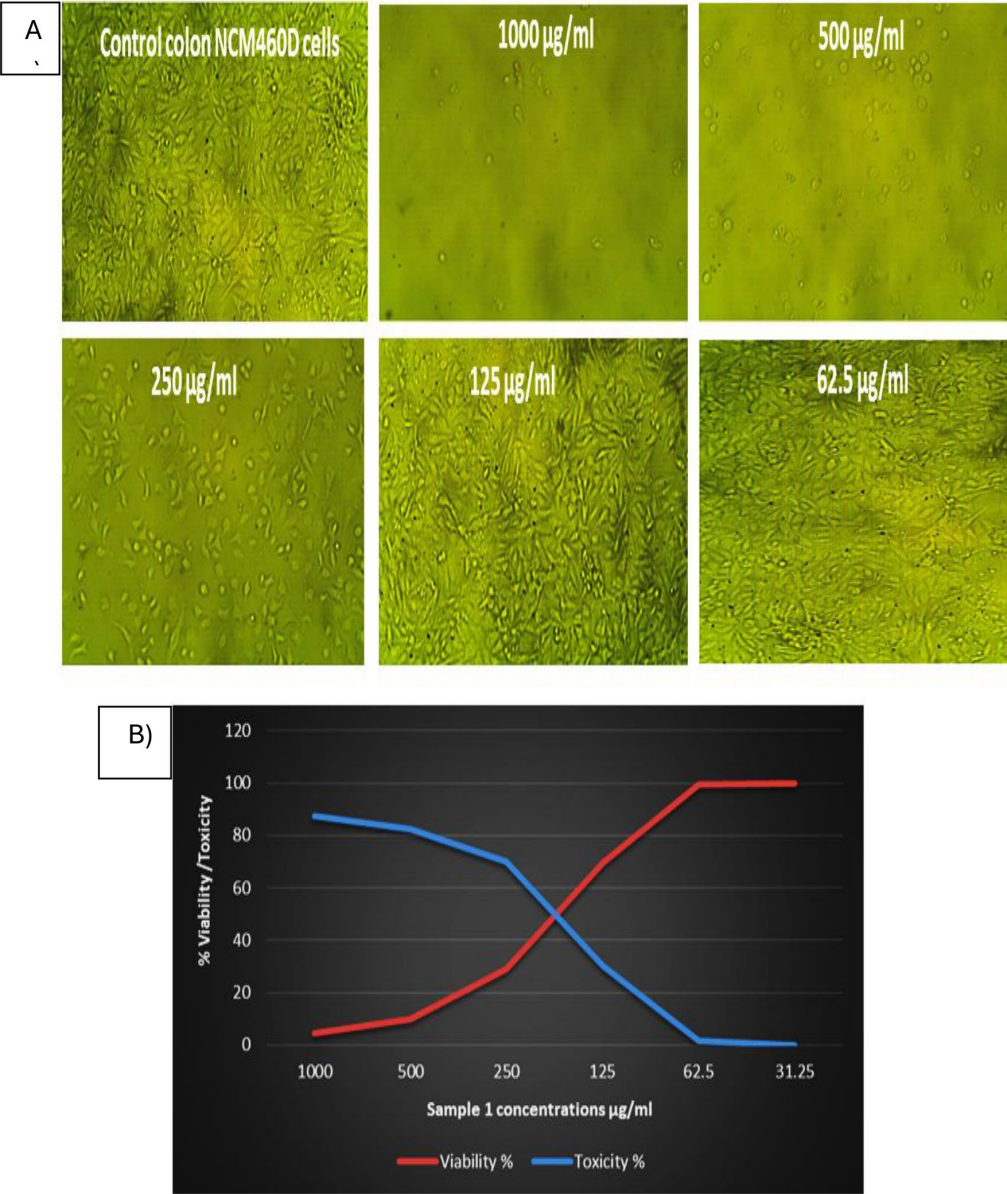
Cell viability is affected by various concentrations of Le-AgNPs. **A** Microscopic images of human colon NCM460D normal cells before and after treatment with different concentrations of Le-AgNPs. **B** Dose-independent curve and IC50 of human colon NCM460D normal cells before and after treatment with different concentrations of Le-AgNPs

The biocompitability of Le-AgNPs on the human colon NCM460D normal cell line was evaluated using the MTT assay. The human colon NCM460D normal cells were exposed to various concentrations ranging from 1000 to 62.5 µg/ml, and cell viability was assessed as shown in Fig. 5A. The MTT assay results demonstrated a dose-dependent decrease in cell viability when exposed to high concentrations of Le-AgNPs. As the concentration of Le-AgNPs increased, a progressive reduction in cell viability was observed. To determine the IC_50_ of Le-AgNPs on human colon NCM460D normal cells, a dose–response curve was obtained. The IC_50_ value represents the concentration of Le-AgNPs at which cell growth was inhibited by 50%. The IC_50_ value of Le-AgNPs on human colon NCM460D normal cells was determined to be 200.53 μg/ml, which indicated that Le-AgNPs have a cytotoxic effect on human colon NCM460D normal cells when the concentrations were higher than the 200.53 μg/ml dose. As shown in Fig. 5B, a decrease in cell count and a concurrent increase in cell apoptosis and debris at concentrations of 1000–250 μg/ml. Whereas, no significant difference was observed between the control group and concentrations of 125 and 62.5 μg/ml. The morphological changes observed in large-scale changes that occur at the cell surface, or in the cytoskeleton, can be followed and related to cell viability. Damage can be identified by large decreases in volume secondary to losses in protein and intracellular ions due to altered permeability to sodium or potassium. Necrotic cells could be observed as nuclear swelling, chromatin flocculation, and loss of nuclear basophilia. On the other hand, apoptotic cells undergo cell shrinkage, nuclear condensation, and nuclear fragmentation.

## Discussion

The process of biofabricating nanoparticles is thought to hold significant potential in impeding the growth of harmful bacteria and fungus on various surfaces and goods. In our investigation, the interaction between the active ingredients in the peel extract of *L. esculentum* and the AgNO_3_ solution led to the creation of AgNP. Many reports indicate that color change is the primary biosynthesis indicator of AgNPs formation (Amr et al. 2023; Farouk et al. 2023).

UV-visible spectrophotometry is used to detect the spectrum of the biosynthesized nanoparticles (Raut et al. 2024). The detected SPR score was in line with other researchers works between 400 and 450 nm (Asif et al. 2022; Alowaiesh et al. 2023; Farouk et al. 2023; Raut et al. 2024). Other than this, an absorption peak at 438 nm showed excellent nanoparticle purity without any other peak as reported by Amr et al. (2023).

The DLS examination showed that the Le-AgNPs particles have size of 186 nm with a polydispersity index (PDI) of 0.578. In the same line, many works report nearly size (Amr et al. 2023; Ansari et al. 2023). DLS analysis gives a comprehensive characteristic regarding the homogeneity of the NPs in colloidal solutions by evaluating the polydispersity index (PDI) values. If the PDI score is more or less than 0.4, the NP’s solution homogeneity is raised or lowered; if the PDI result is equal to or more than 1, the NP’s solution is deemed heterogeneous (Mostafa et al. 2021). According to the current findings, the biosynthesized AgNPs' PDI score was 0.578 confirmed heterogenous nture.

The morphological shape of Le-AgNPs was semi-spherical, with a particle size range of 4.44–27.59 nm. In a study by (Mohamed and Elshahawy, 2022). the average AgNPs size using pomegranate peel extract was 8.0 nm, while using orange peels, the AgNPs were 14.0 nm (Amr et al. 2023). On the other hand, the obtained AgNPs using *Spirogyra hyaline* and *Agaricus bisporus* extract exhibited a spherical morphology with an average grain size of 52.7 and a quasi-spherical shape with a size of 67.45–102.7 nm, respectively (Amr et al. 2023; Farouk et al. 2023).

Because the constructed *L. esculentum* extract comprises active functional groups of the reducing, stabilizing, and capping agents that had gathered on and soured the AgNPs surface, there may have been a variation in the obtained findings of determined size by TEM. The size of the metal nanoparticles indicates how their surface area interacts with the plant extracts; typically, the size of the NPs found through DLS research was greater than that found by SEM, TEM, and XRD investigations. Furthermore, greater NP sizes under the DLS study are associated with the AgNPs metallic core and particle polydispersity (Asif et al. 2022; Weng et al. 2022; Amr et al. 2023; Farouk et al. 2023).

The net charge on the particle's surface was determined using the zeta potential technique. The present study demonstrated that the nonionic nature of Le-AgNPs capping molecules resulted in an AgNPs with a total net charge calculated at -68.44 mv. The findings align with earlier research that indicated the most stable strong zeta potential values for AgNPs were attributed to variations in the distribution of capping polyphenolic ingredients found in the extract (Salem et al. 2020). The extract contains proteins, amino acids, and other necessary metabolites in addition to the biosynthesized NPs, which are responsible for the extract's negative charge (Amr et al. 2023). A long shelf life and great stability were also provided by the negative charge zeta potential, which was discovered to generate repulsion among the AgNPs and inhibit agglomeration and aggregation due to electrostatic stabilization (Ansari et al. 2023).

The FTIR spectra confirmed the results obtained by zeta potential investigation as many functional groups of carboxyl, amine, hydroxyl, and phenolic groups were detected. The *L. esculentum* extract exhibits absorption bands ranging between 3433.60–621.55 cm^−1^, while Le-AgNPs at 3307.64–591.67 cm^−1^. In the line to several studies (Amr et al. 2023; Ansari et al. 2023), the nonionic properties of Le-AgNPs capping molecules caused the vibrational bands to correspond to bonds such C=O, C–C, C–N, C–O–C, C–O–H, and C–Cl, which were in the region range of 3822–600cm^−1^. The interactions between proteins and nanoparticles are well-known to arise from the electrostatic attraction of negatively charged carboxylate groups in enzymes and free amine groups or cysteine residues in proteins (Ansari et al. 2023).

The FTIR analysis results indicate that these functional groups are the capping and reducing agents involved in the synthesis of AgNPs. It confirmed with the previous results that *L. esculentum* is well known for its high levels of vitamins A, B, and C, as well as beta-carotene, and phytosterols (Zia et al. 2017). In addition *L. esculentum* peels are primarily composed of proteins and antioxidants such as phenols, caretonides, leukopines, and glutathione (Carbone et al. 2020). These compounds play a key role in stabilizing and reducing silver salts to form AgNPs.

The XRD located peaks for Le-AgNPs in the distinct positions confirmed the planes (111) and (200) of the face centered cubic FCC structure of the pure crystal synthesized AgNPs matches with the by JCPDS Card No. 36–1451. The peak that was more intensely linked to the (111) plane indicates the AgNPs' primary orientation. The XRD peaks' broadness suggests that the silver particles are nanoscale. The diffracting domains' micro-strains, flattening, and crystallite size all contribute to the peak spreading of XRD lines at half their maximum intensity. Furthermore, compared to the other peaks, the peak intensity along the (111) plane is sharper and more common, suggesting that (111) is the predominant crystallographic plane for AgNPs. The XRD spectra clearly show that no Ag oxide peaks developed, indicating that the phytochemicals in the *L. esculentum* extract effectively capped the biosynthesized AgNPs (Amr et al. 2023). In the same trend (Carbone et al. 2020; Amr et al. 2023) using the bio-reduction method, the XRD result was four peaks at 2θ of 38.4°, 44.5°, 64.90°, and 77.4°, and confirmed the 2θ values are the representation of the following diffraction planes (111), (200), (220), and (311) of the FCC structure of the synthesized AgNPs, which indicated crystalline nature.

The antimicrobial effect of Le-AgNPs showed that the Le-AgNPs had a stronger antibacterial effect against the tested pathogenic bacteria than an antifungal effect against the fungi strains. In a similar study, the antifungal effect of biosynthesized AgNPs with fruit peel extracts reduces the mycelial growth of *A. solani* (Mostafa et al. 2021). AgNPs inhibit *S. aureus*, *S. epidermis*, and *S. pyogens*, as described by AgNPs biosynthesized from cranberries and lingonberries have antibacterial activity against *C. albicans*, *E. coli*, *B. subtilis*, *B. cereus*, *L. monocytogenes*, and *S. typhi* (Puišo et al. 2014; Ansari et al. 2023). According to (Anisa et al. 2020), who investigated the antibacterial impact of AgNPs, *P. aeruginosa* shown the most sensitivity among the examined pathogens, with an inhibition zone diameter (IZD) of 4.8 cm, whereas *B. cereus* and *K. quasipneumoniae* exhibited the highest resistance, with IZDs of 2.0 cm. Furthermore, they disclosed that AgNPs exhibited potent bactericidal properties against G^+ve^ and G^−ve^ microorganisms. Additionally, in the research of (Al-Radadi et al. 2022), *P. aeruginosa* with 18 ± 1.2 mm and *F. solani* with 14.3 ± 0.6 mm showed the highest levels of antibacterial and antifungal activity. In the recent findings of (Said et al. 2024), results appeared that the inhibition zone diameters using Ag-NPs mediated *Lawsonia inermis* extract were 30, 28, 27, 26, 25, 21, and 19 mm for *Acinetobacter* *baumannii*, *K. pneumoniae*, *E. faecalis*, *E. coli*, *P. aeruginosa*, *Proteus mirabilis*, and *S. arlettae*, respectively.

The SEM findings corroborated those of Anisa et al. (2020), who observed that the application of AgNPs caused accumulation on the surfaces of *S. aureus* and *P. aeruginosa* cells, leading to cell invasions or pores that impair bacterial viability. Furthermore, Carlson et al. (2008); Sharma et al. (2022) reported that the microbial cells treated with AgNPs had holes or invasions on the cells as well as damage and distortion at the cell poles brought on by an accumulation of nanoparticles at the cell poles that impacted cell viability and caused cell death. In addition (Al-Radadi et al. 2021) confirmed by membrane damage assay that AgNPs are involved in bacterial cell membrane damage of *P. aeruginosa, B. cereus, S. aureus, and K. pneumoniae*.

One of the most important components of nanosystems' therapeutic usefulness is their biocompatibility. Environmental factors and physico-chemical characteristics of NPs influence their level of biocompatibility (Amr et al. 2023). One of the most commercially viable nanomaterials in the world is AgNPs. Their growing output and market penetration will release AgNP into the environment, intensifying their effects on people and the ecosystem. The cytotoxic effect of Le-AgNPs was studied on human colon NCM460D normal cells. The IC_50_ value was determined to be 200.53 μg/ml. As discussed by (Liu et al. 2010; Hamida et al. 2020), the cytotoxicity effect of nanoparticles is related to their size, irrespective of the coating agent. As (Sharma et al. 2022) discussed, AgNPs with a size 20–50 of 5 nm were more toxic than 20–50 nm particles when using A549, HePG2, MCF-7, and SGC-7901 cell lines. In the same the green-synthesized AgNPs exhibeted high efficacy against the Caco-2 cancerous cell line with IC_50_ of 5.7 ± 0.2 µg/ml (Salem et al. 2020).

In the study of (Sharma et al. 2022) using AgNPs, the IC_50_ value was found to be more than 25 μg/ml on normal human skin fibroblasts (HSF). Also it was reported by Carlson et al. (2008), the smaller nanoparticles with a size of 10–20 nm have a greater cytotoxicity effect than the large particles with 110 nm, as AgNPs with a diameter of 20 nm induce acute neutrophilic inflammation in the lungs of mice. It was explained by Liu et al. (2010) that the cytotoxic effect owing to an increase in Reactive oxygen species (ROS) production using AgNPs. Also, the cytotoxicity effect was explained by Kim and Ryu (2013); Cheng et al. (2021); Said et al. (2024) owing to DNA damage that may be explained by ROS generation. According to our research, AgNPs made from the peel extract of *L. esculentum* are highly biocompatible and might be used in a range of biological applications. Our findings support the biosafety of AgNPs by showing that they are stable in vivo and may have therapeutic uses.

Finally, the study investigates the eco-friendly synthesis of AgNPs using *L. esculentum* peel extract. The biosynthesis was confirmed through a color change of *L. esculentum* extract mixed with silver nitrate solution. The biosynthesized Le-AgNPs were characterized using UV-visible spectroscopy, FTIR spectroscopy, and HR-TEM. The results showed that Le-AgNPs were highly stable and effective against pathogenic bacteria and fungi. They also showed cytotoxicity at an IC_50_ of 200.53 μg/ml on human colon NCM460D normal cells. Thus, AgNPS derived from *L. esculentum* extract may have wide-ranging uses, including antibacterial, anticancer, and wound healing medications, as well as food preservation. Similar findings indicate that biosynthesized nanoparticles that are stabilized, coated, and mediated by bioactive groups utilizing cell-free extracts are more biocompatible, safer, and suitable for use in biomedical applications. Based on our study's findings, it appears that AgNPs made with *L. esculentum peel* extract may find value in medicine and other fields. More research in this area might result in the creation of novel and inventive nanomaterials with a broad variety of possible uses. Also, we suggest more research on AgNP testing for biocontrol biofilms in the food sector.

## Data Availability

All data generated or analyzed during this study are included in this published article. All microbial pathogens were provided by the Agricultural Microbiology Department, Faculty of Agriculture, Ain Shams University, Cairo, Egypt, and was deposited in the following strain providers: *B. subtilis* ATCC 6633 was from ATCC collection: https://www.atcc.org/products/6633.*E. coli* ATCC 8739 was from ATCC collection https://www.atcc.org/products/8739.*K. quasipneumoniae,* ATCC 700603, was from ATCC collection*:*https://www.atcc.org/products/700603.*L. monocytogenes* was deposited in Genbank with gene accession number NC_013768: https://www.ncbi.nlm.nih.gov/nuccore/NC_013768.*S. typhi* DSM 17058 was from the DSM collection: https://www.dsmz.de/collection/catalogue/details/culture/DSM-17058.*S. sonii* DSM 5570 was from the DSM collection: https://www.dsmz.de/collection/catalogue/details/culture/DSM-5570.*A. solani* ATCC 62102, was from ATCC collection: https://www.atcc.org/products/62102.*A. flavus* ATCC 9643 was from ATCC collection: https://www.atcc.org/products/9643.*C. albicans* DSM 1386 was from the DSM collection https://www.dsmz.de/collection/catalogue/details/culture/DSM-1386.*F. oxysporum* ATCC 62506 was from ATCC https://www.atcc.org/products/62506.*R. oryzae* ATCC96382 was from ATCC collection https://www.atcc.org/products/96382.
