# Correction: Compatibility and antimicrobial activity of silver nanoparticles synthesized using *Lycopersicon esculentum* peels

**DOI:** 10.1186/s13568-024-01816-y

**Published:** 2025-01-29

**Authors:** Esraa Ali, Samah H. Abu-Hussien, Esraa Hesham, Shimaa Ahmed, Habiba Mostafa, Ahmed Gamal, Salwa M. El-Sayed, Bahaa Hemdan, Ashraf Bakry, Naglaa M. Ebeed, Hesham Elhariry, Ahmed Galal, Basma T. Abd-Elhalim

**Affiliations:** 1 New Programs, Faculty of Agriculture, Ain Shams University, PO Box 68, Hadayek Shoubra, Cairo, 11241 Egypt; 2 Department of Agricultural Microbiology, Faculty of Agriculture, Ain Shams University, PO Box 68, Hadayek Shoubra, Cairo, 11241 Egypt; 3 Department of Biochemistry, Faculty of Agriculture, Ain Shams University, PO Box 68, Hadayek Shoubra, Cairo, 11241 Egypt; 4 Environmental and Climate Change Research Institute, National Research Center, Giza, 1266 Egypt; 5 Department of Genetics, Faculty of Agriculture, Ain Shams University, PO Box 68, Hadayek Shoubra, Cairo, 11241 Egypt; 6 Department of Food Science, Faculty of Agriculture, Ain Shams University, PO Box 68, Hadayek Shoubra, Cairo, 11241 Egypt; 7 Department of Poultry Production, Faculty of Agriculture, Ain Shams University, PO Box 68, Hadayek Shoubra, Cairo, 11241 Egypt

**Correction to: AMB Express (2024) 14:120**

10.1186/s13568-024-01774-5

In the original publication of this article (Ali et al. 2024), the author “Bahaa Hemdan” was incorrectly denoted as the corresponding author. The only corresponding author of the article is “Basma T. Abd-Elhalim” which has been updated in this correction article. The original article has been updated.
